# Notoginseng Triterpenes Inhibited Autophagy in Random Flaps *via* the Beclin-1/VPS34/LC3 Signaling Pathway to Improve Tissue Survival

**DOI:** 10.3389/fbioe.2021.771066

**Published:** 2021-11-19

**Authors:** Zhiyang Huang, Xiaobin Luo, Yifan Zhang, Yibo Ying, Xiong Cai, Wenjie Lu, Juan Zhao, Yutian Wang, Wenwei Lin, Yurong Tu, Ziyue Xiang, Qiuji Wu, Shengwu Yang, Sipin Zhu, Xiaoyang Li

**Affiliations:** ^1^ Department of Orthopaedics, The Second Affiliated Hospital and Yuying Children’s Hospital of Wenzhou Medical University, Wenzhou, China; ^2^ Department of Orthopaedics, The First Affiliated Hospital of Wenzhou Medical University, Wenzhou, China

**Keywords:** random flap, notoginseng triterpenes, oxidative stress, autophagy, angiogenesis

## Abstract

Random flaps are widely used in tissue reconstruction, attributed to the lack of vascular axial limitation. Nevertheless, the distal end of the flap is prone to necrosis due to the lack of blood supply. Notoginseng triterpenes (NTs) are the active components extracted from *Panax notoginseng*, reducing oxygen consumption and improving the body’s tolerance to hypoxia. However, their role in random flap survival has not been elucidated. In this study, we used a mouse random skin flap model to verify that NT can promote cell proliferation and migration and that increasing blood perfusion can effectively improve the survival area of a skin flap. Our study also showed that the autophagy of random flaps after NT treatment was activated through the Beclin-1/VPS34/LC3 signaling pathway, and the therapeutic effect of NT significantly decreased after VPS34 IN inhibited autophagy. In conclusion, we have demonstrated that NT can significantly improve the survival rate of random flaps through the Beclin-1/VPS34/LC3 signaling pathway, suggesting that it might be a promising clinical treatment option.

## Introduction

Skin flap transplantation is a commonly used method in reconstructive surgery to repair skin loss caused by trauma, surgical resection, and other factors (Schürmann et al., 2009; Lee et al., 2017). Random flaps can be transplanted and arranged arbitrarily because they are not restricted by axial blood vessels and are widely employed in skin flap transplantation (Fang et al., 2020). In the flap, the blood supply is maintained by the vascular network of the pedicle bed of the flap (Lorenzetti et al., 2001). When the flap’s aspect ratio exceeds, the distal region of the flap will necrose due to insufficient blood supply to the microvascular network, which significantly limits randomness (Luo et al., 2021). Therefore, improving the vitality of random flaps and inhibiting necrosis are crucial for improving the clinical application of random flaps.

Notoginseng triterpene (NT) is an active ingredient extracted from *Panax notoginseng,* which has the functions of reducing oxygen consumption of the organism, improving tolerance of the organism to hypoxia, expanding blood vessels and increasing blood flow, and anti-thrombosis and anti-coagulation (Shi et al., 2013; Wang et al., 2021). Studies have shown that NT can be used to treat cerebral infarction and ischemic cerebrovascular disease (Xie et al., 2020). The results have shown that NT has an excellent clinical therapeutic effect on the acute stage of cerebral infarction, and it can significantly improve cerebral edema during global ischemia or focal I/R (Wang et al., 2018). In cytology, morphology, and lipid peroxidation studies, NT has been shown to protect cells from damage caused by energy metabolism disorders (Huang et al., 2015; Huang et al., 2017). Other studies have shown that NT can significantly reduce platelet surface activity; inhibit platelet aggregation, adhesion, and anti-thrombosis; improve microcirculation; and other effects (Wang et al., 2016). However, it has not been examined yet if it can enhance the survival of voluntary skin flaps. Autophagy is referred to as the rough-surfaced endoplasmic reticulum of the ribosome area of the double-membrane package, which is part of the cytoplasm and cell organelles. Protein compositions, such as the degradation of domestic demand autophagy, will form, and fusion with lysosomes that produce autophagy-lysosome and degradation of the contents of the package to realize the metabolism of the cell itself and some organelles needs to be updated (Hale et al., 2013; Hurley and Young, 2017; Doherty and Baehrecke, 2018; Yu et al., 2018). In this process, Beclin-1 is an essential molecule which plays a key role in the formation of autophagosomes, that can mediate the localization of other autophagic proteins to phagocytes and thus regulate the formation and maturation of autophagosomes (Menon and Dhamija, 2018; Xu and Qin, 2019; Kaur and Changotra, 2020). In an acute injury, autophagy can clear damaged organelles and convert them into energy (Tamargo-Gómez and Mariño, 2018; Yang et al., 2019; Lin et al., 2021). Therefore, moderately upregulated autophagy expression after acute injury is beneficial to tissue survival. Apoptosis is activated at the distal end of random flaps, autophagy is inhibited due to prolonged injury time, and the tissue self-protection ability is weakened. Therefore, the activation of autophagy in random flaps is essential (Chen et al., 2017; Li J. et al., 2021; Tu et al., 2021).

In this study, we comprehensively investigated the application of NT in promoting angiogenesis and antioxidant stress of random flaps and found that NT can improve the survival rate of random flaps by promoting autophagy. The VPS34 IN inhibition experiment showed that NT promoted the survival of random flaps through the Beclin-1/VPS34/LC3 pathway.

## Materials and Methods

### Reagents and Antibodies

NT, 3-methyladenine (3MA), and VPS34 IN were supplied by MedChemExpress (NJ, United States). The NT sample was dissolved in normal saline (Xie et al., 2019). Primary antibodies to Beclin-1, VPS34, LC3B, P62, CD34, SOD1, eNOS, and HO-1 were bought from Abcam (Cambridge, United Kingdom). Primary antibodies to VE-cadherin and VEGF were supplied by Affinity Biosciences (OH, United States) and primary antibodies to LC3 I/II were bought from ZenBio (Chengdu, China). Anti-mouse and anti-rabbit secondary antibodies IgG-conjugated with Alexa Fluor 488 (green) and Alexa Fluor 594 (red), or with horseradish peroxidase (HRP), were obtained from Abcam (Cambridge, United Kingdom). Umbilical vein endothelial cells were bought from OTWO (Shenzhen, China). RPMI 1640 medium (1640) and fetal bovine serum (FBS) were bought from Gibco (California, United States).

### Cell Culture and Cell Stimulate

Umbilical vein endothelial cells (UVECs) were cultured in RPMI 1640 complete medium containing 10% serum. The cells were divided into four groups: the control group, the H_2_O_2_ group (added 100 μM H_2_O_2_), the NT group (added 100 μM H_2_O_2_ + 100 μg/ml NT), and the NT + VPS34 IN group (added 100 μM H_2_O_2_ + 100 μg/ml NT + 10 nM VPS34 IN).

### Cell Scratch Assay and CCK-8 Test

The UVECs were laid on a 12-well petri dish and cultured overnight until a fused monolayer was formed. The cells were scratched with a 200-μL pipette and washed with phosphate-buffered saline (PBS) three times. The images of monolayers of injured cells were captured using light microscopy at 0, 6, 12, and 24 h post-injury. UVECs were laid onto 96-well plates with 10,000 cells per well. After 12 h, the cells were divided into three groups: the control group, the 50 μg/ml NT group, and the 100 μg/ml NT group. The CCK-8 kit was used to detect cell proliferation.

### Animal Model of the Random Flap

Sixty healthy C57BL/6 male mice (20–22 g) were provided by the Experimental Animal Center of Wenzhou Medical University. The animal experiment was approved by the Animal Protection and Use Committee of Wenzhou Medical University (Wydw. 2021-0242). These mice were randomly divided into 4 groups, including the control group, NT-treated group (NT group), NT+3MA treated group (NT+3MA group), and NT + VPS34 IN–treated group (NT + VPS34 IN group). Prior to surgery, all mice were anesthetized by 1% sodium pentobarbital (50 mg/kg, i.p.). The random dorsal flap was constructed by removing all the blood-supplying arteries from the central back of the mouse with an aspect ratio of 8:3. Finally, the detached flap was fixed. The mice were killed on the seventh day after surgery. After euthanasia, the skin flap tissue and substantial organs were immediately collected for follow-up experiments.

### Treatment Protocols

The mice in the NT and NT+3 MA groups were treated with 40 mg/kg/d NT intraperitoneal injection for 7 days and the control group was treated with an unequal volume of saline. The mice in the NT+3MA group received 15 mg/kg/d 3MA 30 min before NT through intraperitoneal injection. The mice in the NT + VPS34 IN group received 5 mg/kg/d VPS34 IN 30 min before NT through intraperitoneal injection.

### General Evaluation of Flap Survival

Three and seven days after surgery, the survival of the flap was observed using high-quality photography. The macroscopic development, appearance, and color characteristics of the flaps were observed 7 days after surgery. All images were measured using ImageJ (MD, United States). To calculate the survival area, the survival area percentage was measured as follows: survival area range/total area × 100%.

### Tissue Edema Assessment

Seven days after surgery, the skin flaps were collected from each group and weighed. Then, they were dehydrated until the weight remained stable. The degree of edema can be determined as follows: ([wet weight − dry weight]/wet weight) × 100%.

### Laser Doppler Blood Flow Imaging

LDBF imaging was used to determine the blood supply and vascular flow of the flap. Under anesthesia, the mouse lies in the prone position in the scanning area, and the laser Doppler imager scans the entire flap area. The blood flow at 0, 3, and 7 days was observed postoperatively through the color of the live blood flow images provided. The quantification of blood flow was performed via perfusion units, and the blood flow was measured using moorLDI Review software (version 6.1).

### Hematoxylin and Eosin and Masson’s Trichrome Staining

Tissue samples were taken from each group for pathologic analysis. The tissue was fixed with 4% paraformaldehyde and embedded in paraffin, cut into 5 μm for H and E and Masson’s trichrome staining. H and E–stained sections were examined under a light microscope (x 10) to assess histological changes, including granulation tissue, swelling, and microvascular remodeling. Masson-stained sections were examined under a light microscope (x 10) to assess fibrotic accumulation.

### Immunofluorescence

The primary antibodies against LC3B (1:500), P62 (1:1,000), CD34 (1:300), eNOS (1:200), and SOD1 (1:200) were used. Next, the tissues were washed with PBS three times. Then, the corresponding secondary antibodies (1:1,000) were applied for 1 h. The tablets were washed three times with PBST and were finally sealed with a sealing solution containing DPAI. A Nikon ECLIPSE Ti microscope (Nikon, Tokyo, Japan) was used to take images. Single dyeing uses grayscale display and double dyeing uses color display.

### Western Blot Analysis

The extracted protein was determined by Coomassie bright blue method, and the protein sample was prepared with a concentration of 40 μg/20 μLl. After electrophoresis, the proteins were transferred onto polyvinylidene fluoride (PVDF) membranes (Bio-Rad, Hercules, CA, United States). The membrane was pre-incubated with 5% milk (Bio-Rad) for 2 h and incubated with each of the following antibodies: anti–Beclin-1 (1:500), anti-VPS34 (1:1,000), anti-LC3 I/II (1:1,000), anti-P62 (1:10,000), anti–HO-1 (1:1,000), anti-SOD1 (1:3,000), anti-eNOS (1:2000), anti-VEGF (1:500), anti–VE-cadherin (1:500), anti-CD34 (1:500), and GAPDH (1:3,000) at 4°C for 12 h. After that, the membranes were washed with TBST three times and incubated with secondary antibodies for 90 min. The signals were detected using ChemiDoc XRS + Imaging System (Bio-Rad).

### Blood Biochemical Test

The blood of mice was collected by anticoagulant vessels containing heparin lithium, and the supernatant plasma was collected after centrifugation at 3,000 r at 4°C for 5 min. 100 μL of plasma was poured into a blood biochemistry tray (Micro-nano Chip, Tianjin, China) and diluted with 430 μL of pure water. An M3 biochemical analyzer was used (Micro-nano Chip, Tianjin, China) to test related indexes on the test tray with better density.

### Statistical Analysis

Statistical analysis was performed using a two-sided Student’s t-test. SPSS13 was used for statistical analyses. Data were expressed as mean ± standard deviation (SD). *p* < 0.05 was considered statistically significant.

## Results

### NT Could Promote Cell Proliferation and Migration

**FIGURE 1:**
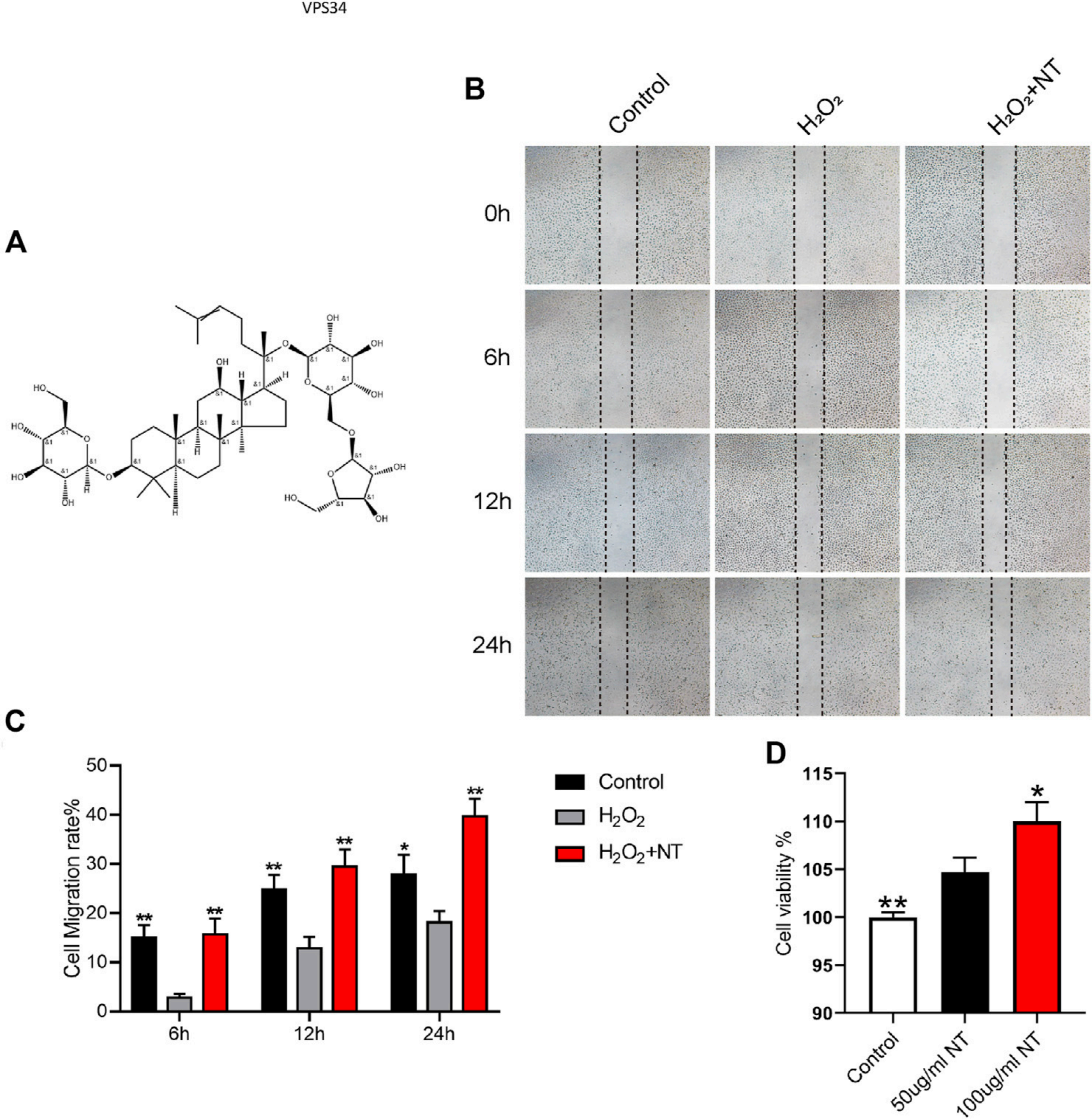
NT promotes cell migration by inhibiting oxidative stress. **(A)** Structure illustration of NT. **(B)** Images of wound healing in the control, H_2_O_2_, and H_2_O_2_+NT groups. Images were captured at 0, 6, 12, and 24 h after the scratch. **(C)** Cell migration rate that were affected by the abovementioned factors for 6, 12, and 24 h. **(D)** Cell viability of each cell. Data were expressed as mean ± SD, *n* = 3. “**” and “*” represent *p* <0.01 or *p* <0.05 versus the H_2_O_2_ and 50 ug/mL NT groups, indicating statistical significance.

**FIGURE 2:**
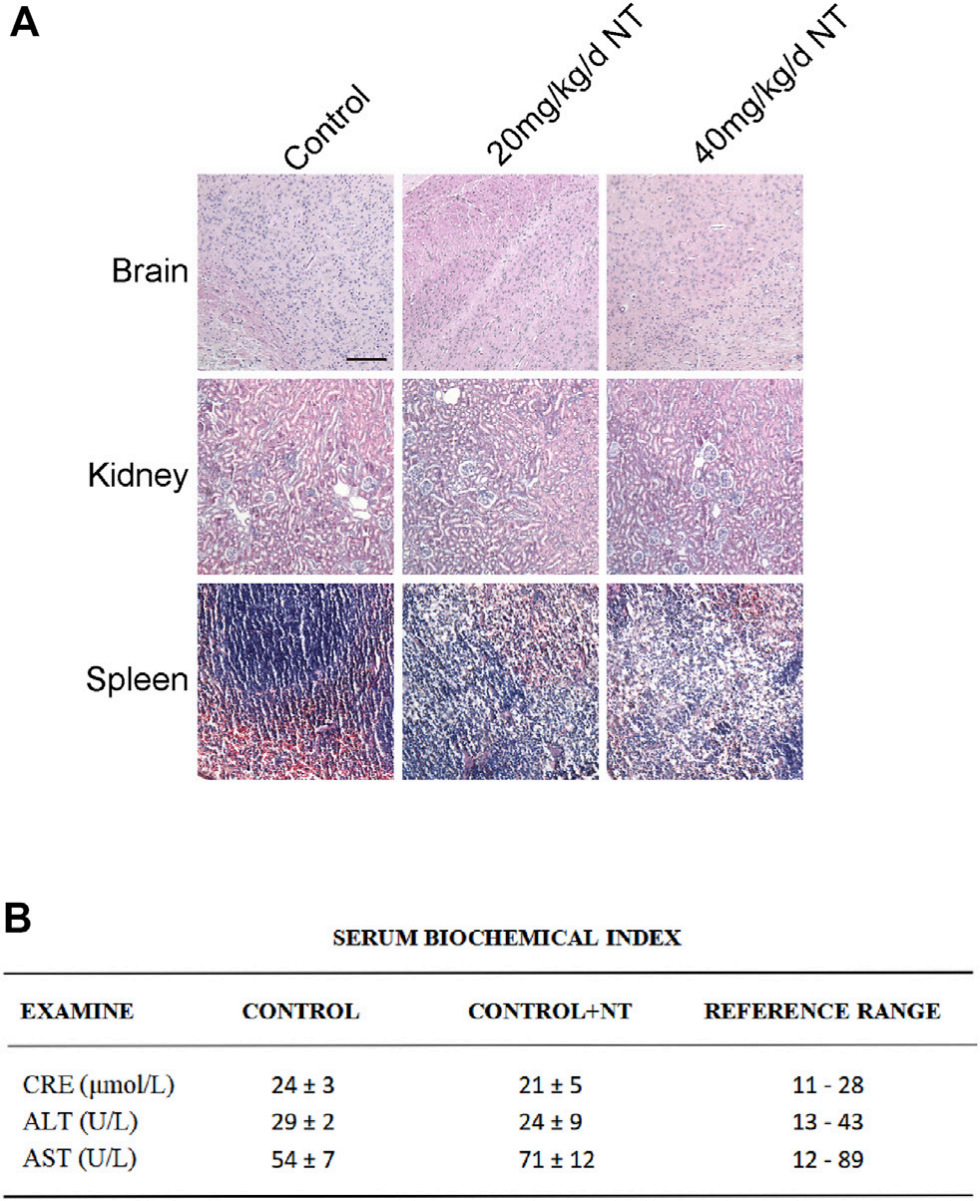
Tissue toxicity verification of NT. **(A)** H and E staining of the brain, kidneys, and spleen of mice in each group. Magnification: 20X; Scale bar = 100 μm. (B) Blood biochemical test in each group. Data were expressed as mean ± SD, n = 3. n.s. represents no statistical significance.

NT is the active extract of *Panax notoginseng* (Figure 1A), which can promote blood circulation, remove blood stasis, and increase blood flow. The proliferation, migration, and remodeling of vascular endothelial cells are crucial in random flap transplantation. We first tested the migration ability of UVECs by the cell scratch test, and the experimental results showed that the migration ability of UVECs was significantly decreased after H_2_O_2_ stimulation than the control group, and the migration ability of UVECs was well-recovered after NT treatment (Figures 1B–C). Then, we used the CCK-8 test to detect UVEC proliferation by NT, and the experimental results showed that NT could effectively promote the proliferation of UVECs (Figure 1D). Furthermore, we detected the toxicity of NT *in vivo*, and no obvious pathologic changes were found in the brain, kidneys, spleen, and other tissues by H and E staining (Figure 2A). The fluctuation of AST, ALT, and CRE in blood biochemistry was found to be within a normal range (Figure 2B). In conclusion, NT can promote cell proliferation and migration with minimum toxicity.

### NT Could Improve the Survival Rate of Random Flaps

**FIGURE 3:**
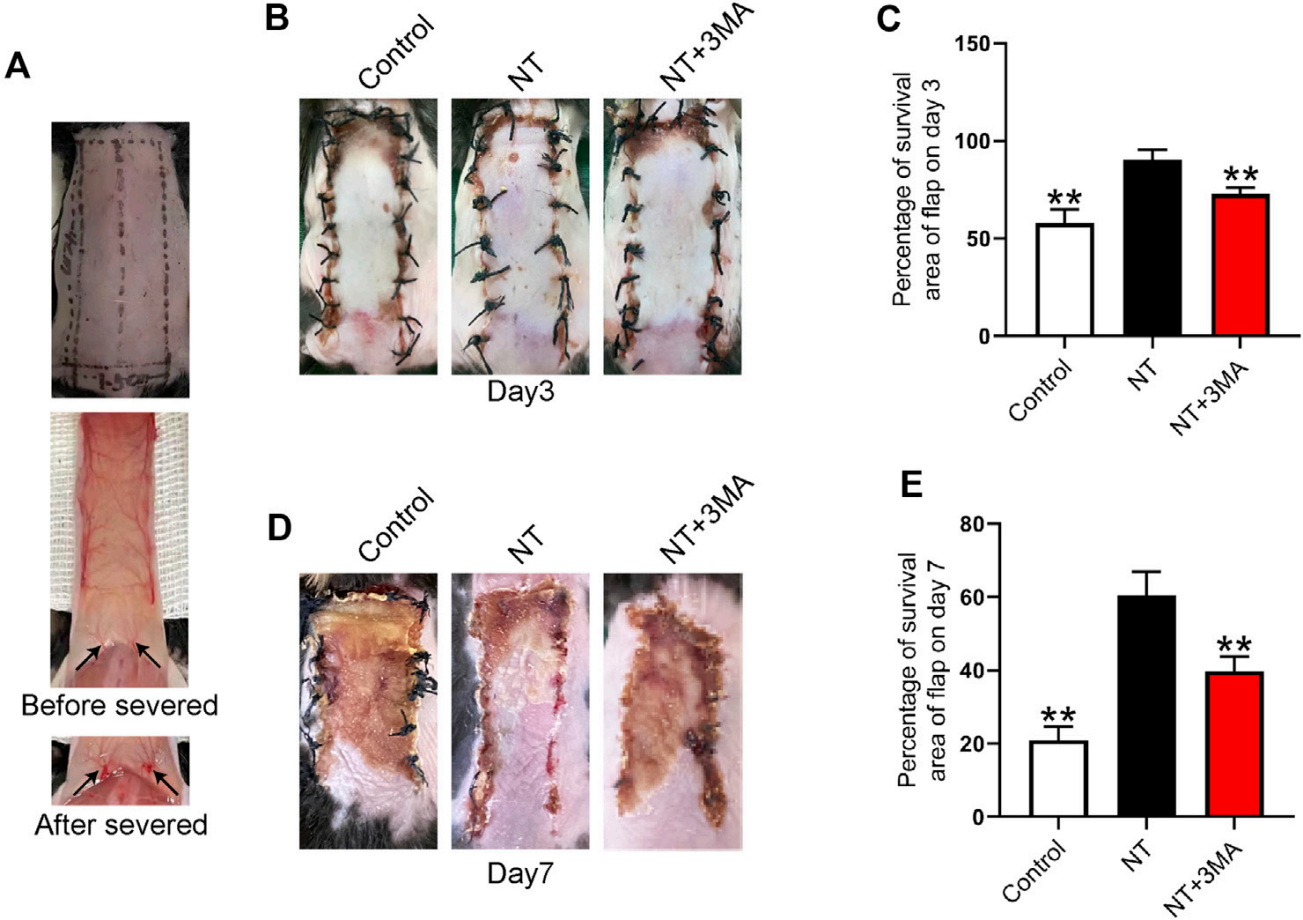
Application of NT directly increased the storage area of the flaps. **(A)** Photographs of the flap modeling area and visual images before and after vascular occlusion. **(B,D)** Images reflecting flap survival at the third and seventh day after operation in the control group, treatment group with NT, and treatment group with NT+3MA. **(C,E)** Chart of the percentage of the skin flap stock area in each group for 3 and 7 days. Data were expressed as mean ± SD, n = 3. “**” represents *p* <0.01 versus the NT group, indicating statistical significance.

The distal end of the random flap is prone to ischemic necrosis because the pedicle of a blood vessel is cut off during modeling (Figure 3A). We first observed the necrosis of the flap and quantified the necrotic area. Our results showed that small areas of necrosis began to occur in the flaps of each group 3 days after surgery, and the survival of the flap in the control group was less than that in the NT and NT+3MA groups (Figures 3B–C). The flap survival rate in the NT group was higher than that in the NT+3MA group. On the seventh postoperative day, a large area of skin flap necrosis occurred in both the control and NT+3MA groups, while the survival rate of the NT group was better than that of the other two groups (Figures 3D–E). Our results suggest that NT can effectively promote the survival of random flaps, and the pro-survival effect may be related to autophagy activation.

### NT Could Enhance the Blood Flow Signal in Random Flaps

**FIGURE 4:**
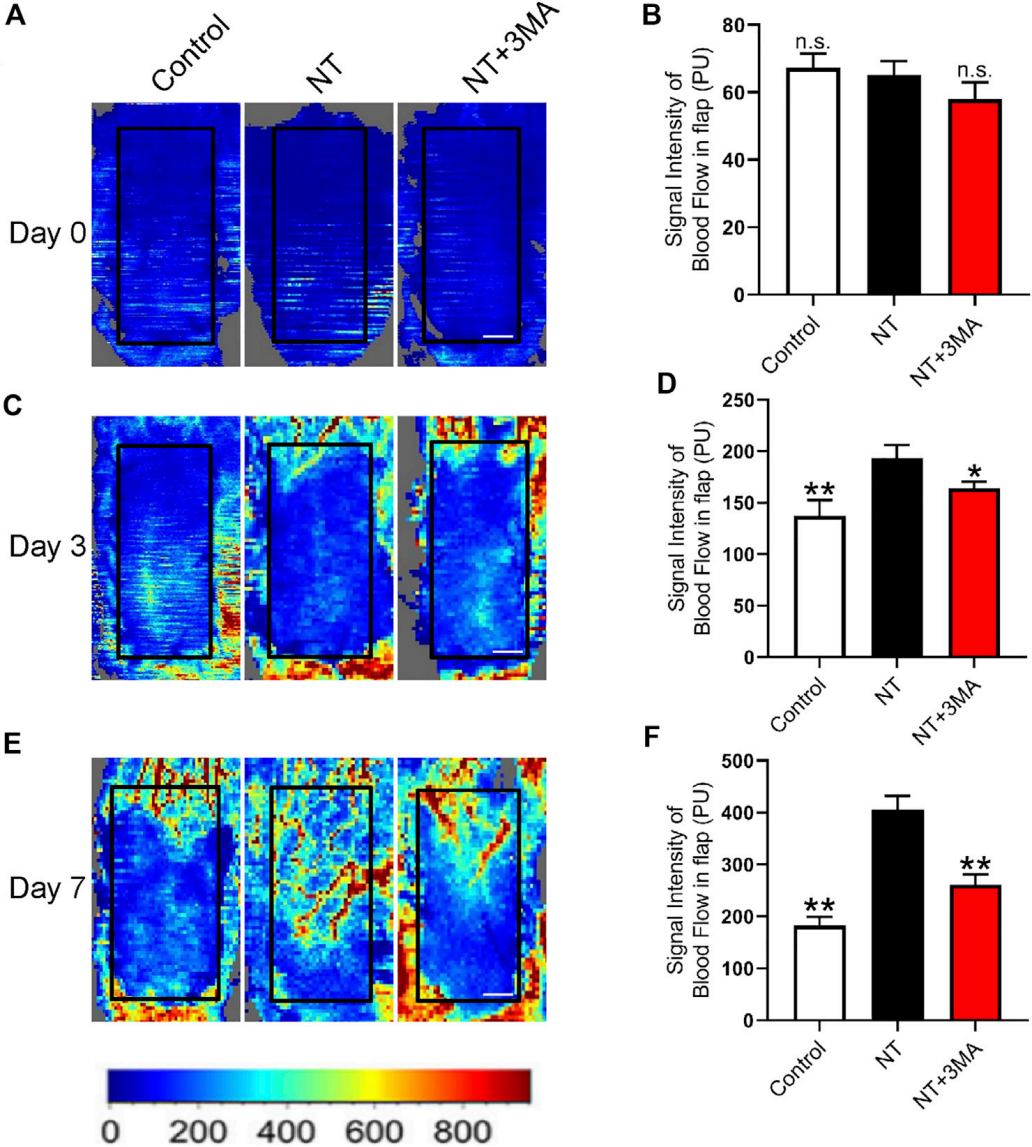
NT promotes blood flow reconstruction of the skin flap. **(A)** LDBF technique shows the subcutaneous blood flow status immediately after surgery, as well as 0 days. (**B)** Statistical result of blood flow signal intensity at 0 days. **(C)** LDBF technique shows the subcutaneous blood flow status immediately after surgery, as well as three days. **(D)** Statistical result of blood flow signal intensity at the third day. **(E)** LDBF technique shows the subcutaneous blood flow status immediately after surgery, as well as the number of days. **(F)** Statistical result of blood flow signal intensity at the seventh day. Scale: 0.5 cm. Data were expressed as mean ± SD, *n* = 3. “**” and “*” represent *p* <0.01 or *p* <0.05 versus the NT group, indicating statistical significance. n.s. represents no statistical significance.

Adequate blood perfusion is the key to flap survival, and LDBF was used to trace the microvascular network reconstruction. Our results showed that the random flaps in each group lost blood supply after modeling, and the blood flow signal in the NT and NT+3MA groups rebounded on the third day after surgery, as well as the blood flow signal in the NT group was higher than that in the NT+3MA group (Figures 4A–D). On postoperative day 7, blood flow signals in the NT group showed significant recovery among the three groups. The control and NT+3MA groups showed no significant recovery compared to the third postoperative day (Figures 4E,F). In conclusion, NT can effectively restore random flap perfusion and promote flap survival.

### NT Could Improve the Blood Perfusion and Tissue Morphology of Random Flaps

**FIGURE 5:**
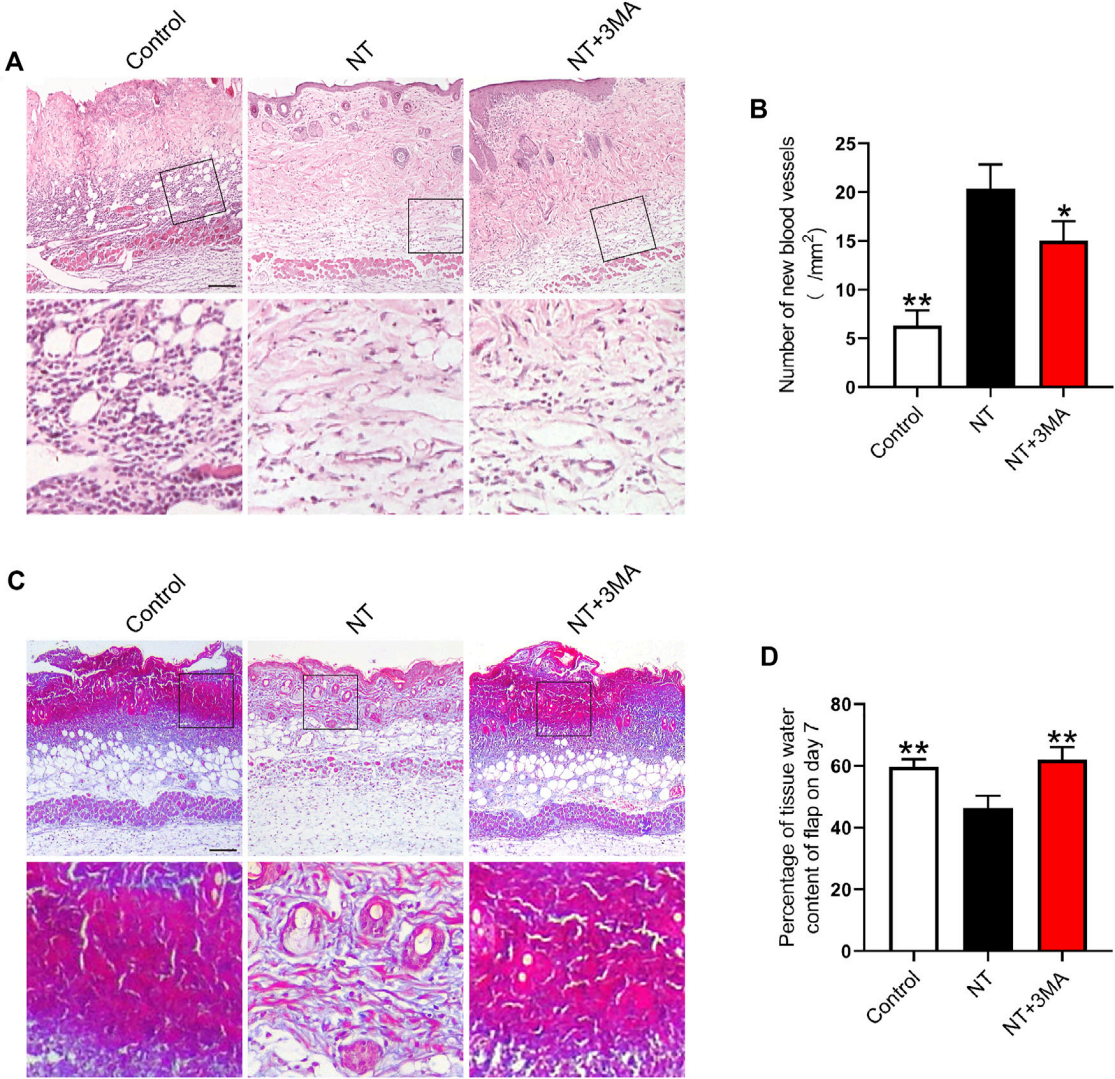
Application of NT results in a better histologic state of the flap. **(A)** H and E staining images of the control, NT, and NT+3MA groups. Magnification was × 10, Scale bar = 200 μm. Area selected by the white border is taken at high magnification as the representative image, Scale bar = 50 μm. **(B)** Number of new blood vessels. **(C)** Masson’s trichrome stain images of each group showing blood vessels and collagen fibers. Scale bar = 200 μm. High-power images were also equipped, Scale bar = 50 μm. **(D)** Percentage of the tissue water content of the flap on day 7. Data were expressed as mean ± SD, *n* = 3. “*” and “**” represent *p* <0.05 or *p* <0.01 versus the NT group.

Neovascularization is the key to increase blood perfusion. We used histologic staining to observe the blood supply regeneration of random flaps. H and E staining showed no obvious neovascularization in the flap of the control group. On postoperative day 7, the NT group showed a large number of microvessels. The number of microvessels in the NT+3MA group was less than those of the NT group (Figures 5A,B). Masson’s trichrome staining showed that the NT group had angiogenesis and collagen fiber arrangement was dense and in order, while the collagen fiber arrangement was irregular and loose in the control and NT+3MA groups (Figure 5C). Then, we assessed the degree of edema in the flap, and the results showed that the degree of edema in the NT group was lower than that in the control and NT+3MA groups (Figure 5D). In conclusion, NT could effectively improve the blood perfusion and tissue morphology of random flaps to promote the survival of flaps.

### NT Could Promote Angiogenesis and Inhibit Oxidative Stress

**FIGURE 6:**
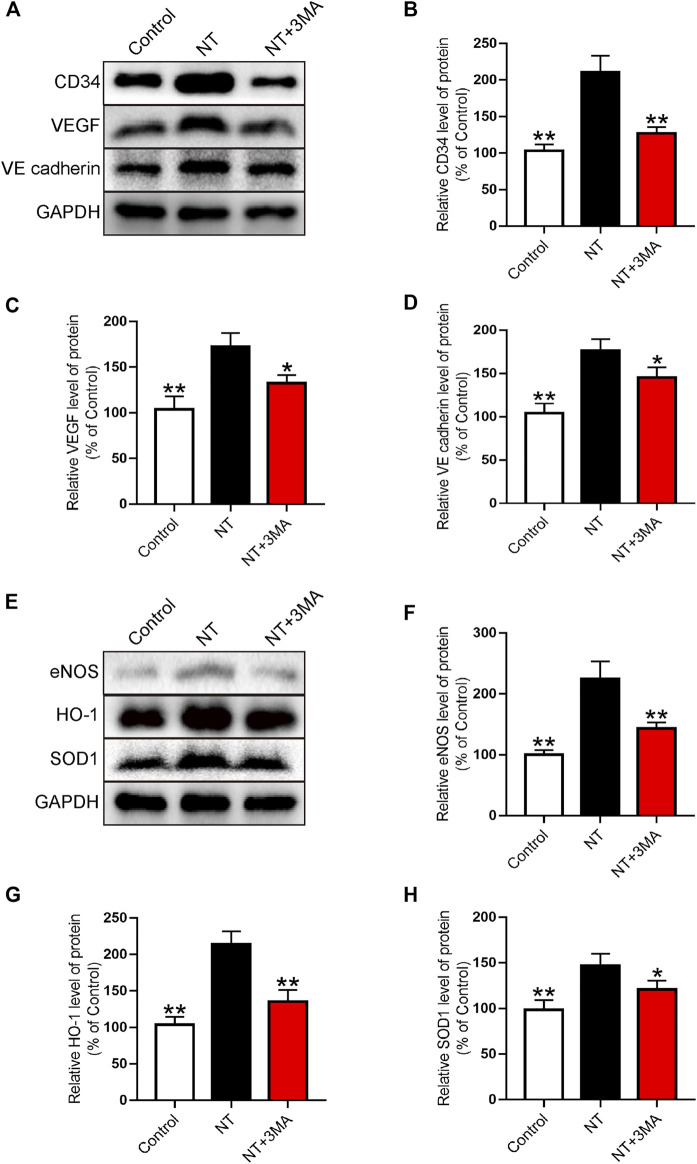
NT promotes blood vessel proliferation and reduces the level of oxidative stress **(A)** Western blot results showing the expression of CD34, VEGF, and VE-cadherin in different groups. **(B–D)** Quantitative analysis of CD34, VEGF, and VE-cadherin protein expressions. **(E)** Western blot results showed the expression of eNOS, HO-1, and SOD1 in different groups. **(F–H)** Quantitative analysis of eNOS, HO-1, and SOD1 protein expressions. Data were expressed as mean ± SD, *n* = 3. “**” and “*” represent *p* <0.01 or *p* <0.05 versus the NT group, indicating statistical significance.

Western blotting was used to further analyze the expression of neovascularization. Our results showed that the CD34 index representing neovascularization significantly increased after NT treatment, and the number of neovascularization in the NT+3MA group was significantly reduced than that in the NT group (Figures 6A,B). The expression levels of VEGF promoting angiogenesis and VE-cadherin promoting vascular maturation were also significantly increased in the NT group compared with those of the control group. The levels of VEGF and VE-cadherin in the NT+3MA group were significantly lower than those in the NT group (Figures 6A,C,D). Then, the oxidative stress levels of random flaps were analyzed by Western blotting. We detected eNOS, HO-1, SOD1, and other reductive indexes and found that the expression of reductive indexes in the NT+3MA group was significantly higher than that in the control group after NT treatment, and the expression of reductive indexes in the NT+3MA group was significantly lower than that in the NT group (Figures 6E–H). In summary, NT can effectively promote angiogenesis, maintain the stability of regenerated blood vessels, and promote vascular recanalization to form an arteriovenous loop. Moreover, it can promote the generation of reducing substances to reduce the damage caused by oxidative stress.

### NT Promoted the Survival of Random Flaps by Activating Autophagy

**FIGURE 7:**
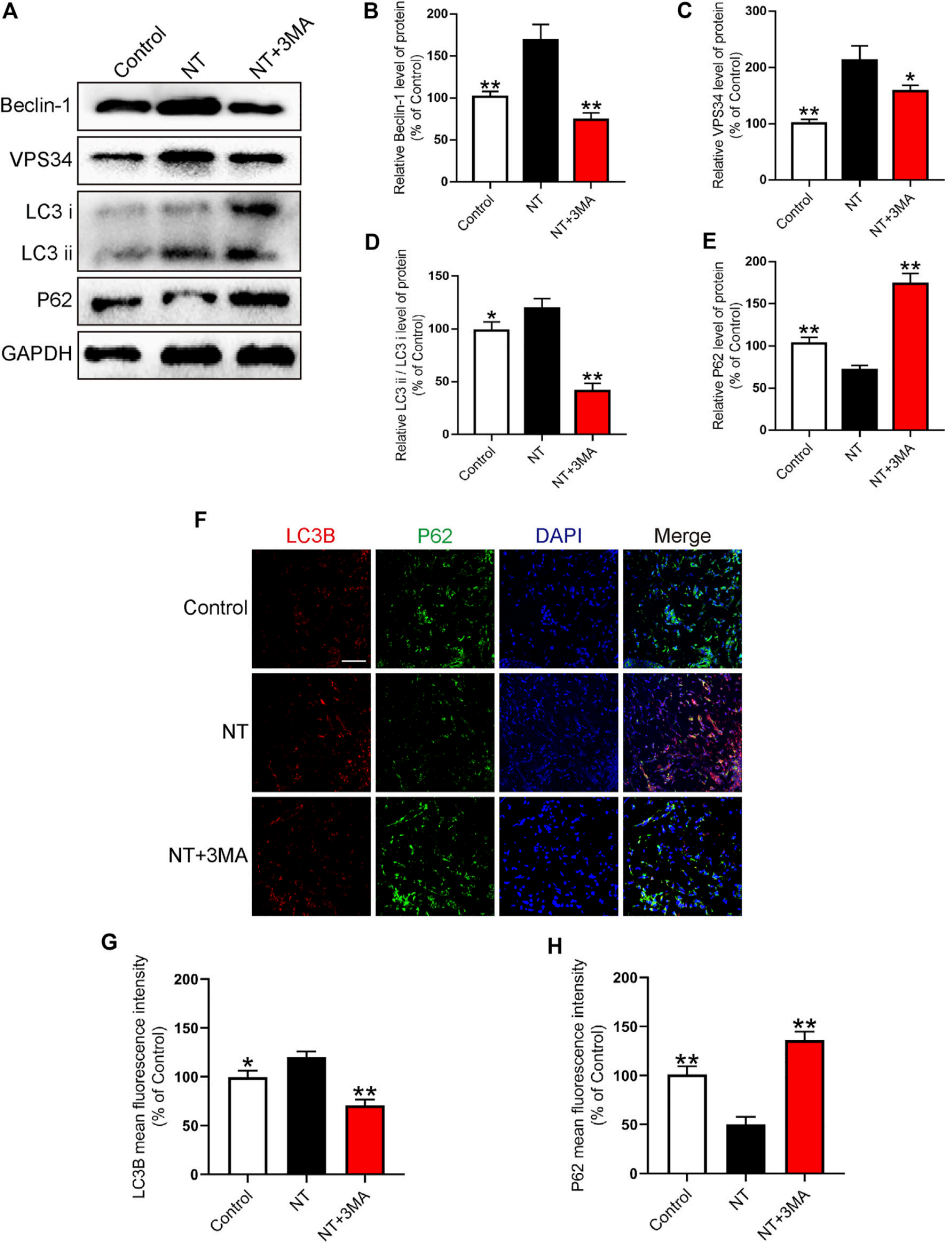
NT improves autophagy levels **(A)** Western blot results showing the expression of Beclin-1, VPS34, LC3, and P62 in different groups. **(B–E)** Quantitative analysis of Beclin-1, VPS34, LC3, and P62 protein expressions. **(F)** Immunofluorescence showing the expression of LC3B (red), P62 (green), and DAPI (blue) in different groups. Magnification: 20X; Scale: 100 μm. **(G–H)** Mean fluorescence intensity of LC3B and P62 in different groups. Data were expressed as mean ± SD, *n* = 3. “**” and “*” represent *p* <0.01 or *p* <0.05 versus the NT group, indicating statistical significance.

In random flaps, autophagy is needed to generate energy to supply cell survival due to the lack of vascular pedicle and tissue blood supply. Therefore, the activation of autophagy can effectively promote the survival of flaps. We first detected autophagy-related proteins, namely, Beclin-1, VPS34, LC3, and P62 using Western blotting. We found that the autophagy activation indexes, namely, Beclin-1, VPS34, and LC3 in the NT group were significantly higher than those in the control group. Beclin-1 in the NT+3 MA group was significantly higher than that in the control group after autophagy inhibitor 3MA. The expression levels of VPS34 and LC3 were significantly downregulated (Figures 7A–D). P62 as a substrate for autophagy decreased significantly in the NT group than in the control group. The level of P62 in the NT+3MA group was significantly higher than that in the NT group (Figures 7A,E). Then, we further verified the expression of LC3B and P62 in each group by immunofluorescence. Our results showed that LC3B in the NT group was significantly higher than that in the control group, and the LC3B level in the NT+3MA group was significantly lower than that in the NT group. The results of P62 are exactly the opposite of those of LC3B (Figures 7F–H). In summary, NT promotes the survival of random flaps through the Beclin-1/VPS34/LC3 pathway.

### NT Inhibits Autophagy Through the Beclin-1/VPS34/LC3 Pathway

**FIGURE 8:**
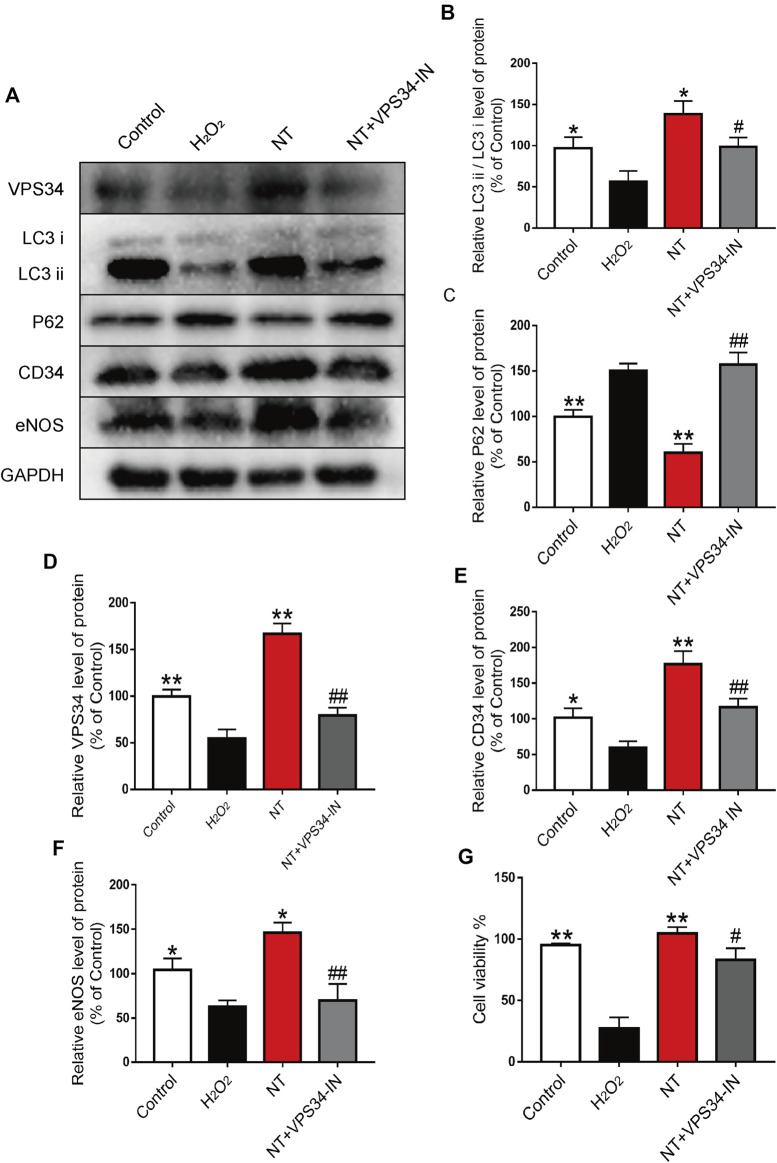
NT regulates autophagy through the Beclin-1/VPS34/LC3 pathway *in vitro* **(A)** Western blot results showing the expression of VPS34, LC3, P62, CD34 and eNOS in different groups. **(B–F)** The quantitative analysis of VPS34, LC3, P62, CD34 and eNOS protein expression. **(G)** Cell viability of each cell. The data were expressed as the mean ± SD, n = 3. “**” and “*” represent *p*<0.01 or *p*<0.05 versus the NT group, “##” and “#” represent *p*<0.01 or *p* 0.05 versus the NT + VPS34 IN group, indicating statistical significance.

**FIGURE 9:**
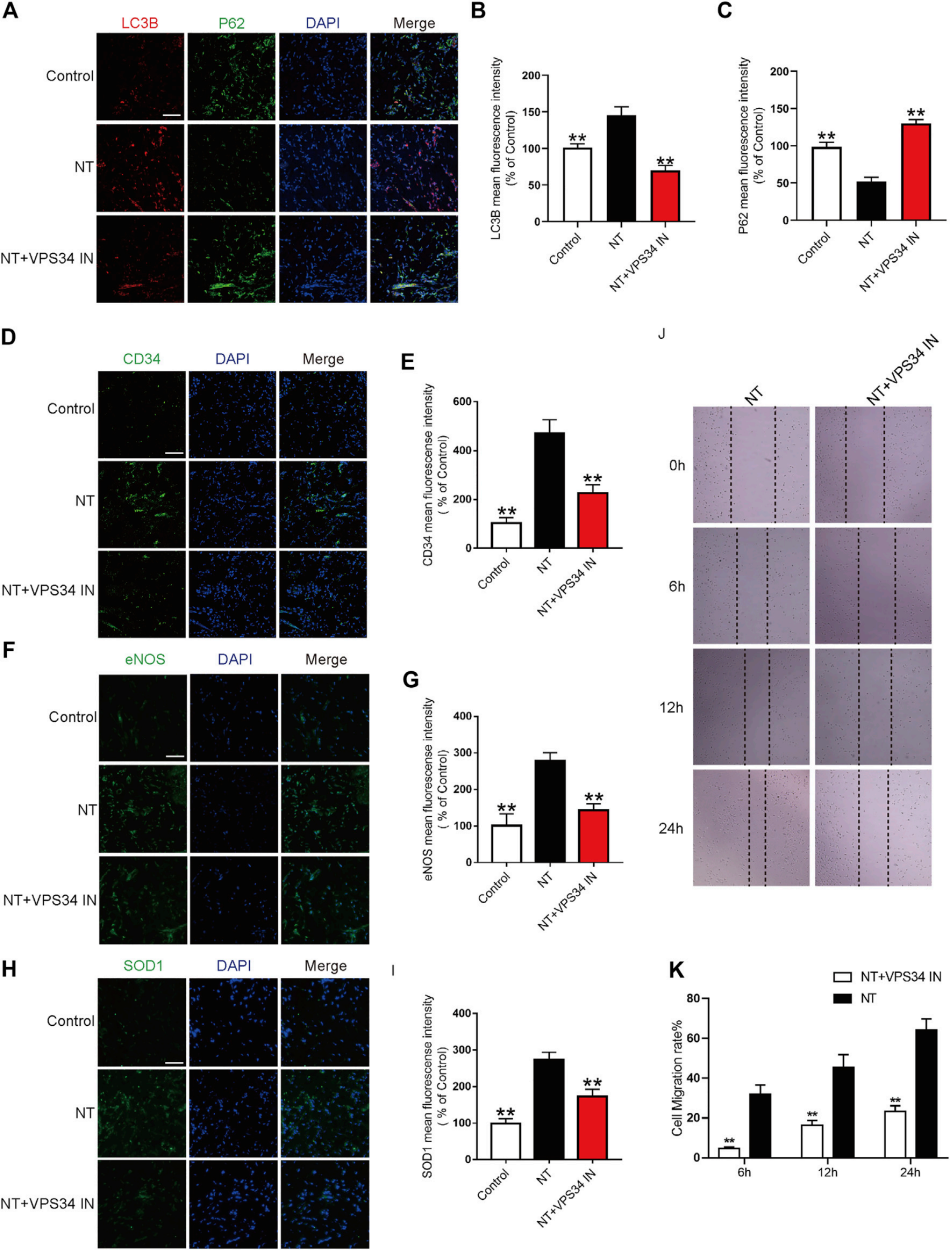
NT regulates autophagy through the Beclin-1/VPS34/LC3 pathway *in vitro* **(A)** Immunofluorescence showing the expression of LC3B (red), P62 (green), and DAPI (blue) in different groups. Magnification: 40X; Scale bar: 50 μm. **(B–C)** Mean fluorescence intensity of LC3B and P62 in different groups. **(D)** Immunofluorescence showing the expression of CD34 (green) and DAPI (blue) in different groups. Magnification: 40X; Scale bar: 50 μm. **(E)** Mean fluorescence intensity of CD34 in different groups. **(F)** Immunofluorescence showing the expression of eNOS (green) and DAPI (blue) in different groups. Magnification: 40X; Scale bar: 50 μm. **(G)** Mean fluorescence intensity of eNOS in different groups. **(H)** Immunofluorescence showing the expression of SOD1 (green) and DAPI (blue) in different groups. Magnification: 40X; Scale bar: 50 μm. **(I)** Mean fluorescence intensity of SOD1 in different groups. **(J)** Images of wound healing in the NT and NT + VPS34 IN groups. Images were captured at 0, 6, 12, and 24 h after the scratch test. **(K)** Cell migration rate that was affected by the abovementioned factors for 6, 12, and 24 h. Data were expressed as mean ± SD, *n* = 3. “**” and “*” represent *p* <0.01 or *p*<0.05 versus the NT group, “##” and “#” represent *p* <0.01 or *p* <0.05 versus the NT + VPS34 IN group, indicating statistical significance.

To verify the autophagy pathway of NT action, the VPS34 inhibitor: VPS34 IN was first used to inhibit the expression of VPS34 at the cellular level. Western blotting results showed that the expression of VPS34 activated by NT was downregulated after VPS34 IN, LC3 expression was also downregulated, and P62 expression was increased. The expression levels of CD34, an indicator of angiogenesis, and eNOS and SOD1, an indicator of oxidative stress protection, were down-regulated after using VPS34 IN (Figures 8A–F). CCK-8 results showed that VSP34 IN significantly inhibited the protective effect of NT on UVECs (Figure 8G). Next, we used immunofluorescence to detect the expression of autophagy, angiogenesis, and oxidative stress protectants in tissues. Our results showed that VPS34 IN inhibited autophagy, reduced angiogenesis, and downregulated the expression of oxidative stress–protective substances (Figures 9A–I). The cell scratch assay showed that VPS34 IN could significantly inhibit the migration activity of UVECs (Figures 9J,K). In conclusion, NT activates autophagy in random flaps through the Beclin-1/VPS34/LC3 pathway, promoting the survival of flaps.

## Discussion

During flap repair, avascular necrosis of the distal flap is the most common cause of surgical failure (Bai et al., 2021; Zhang et al., 2021). Therefore, the most important factor in protecting flap survival is to promote angiogenesis (Zhu X. et al., 2021; Lou et al., 2021). However, the adverse effects of I/R injury and nutrient deficiency during angiogenesis should not be ignored (Basu et al., 2014; He et al., 2021). Our findings showed that NT was the core factor for the survival of random flaps. NT promoted the survival of random flaps by inhibiting angiogenesis and inhibiting oxidative stress and also improved the tolerance of random flaps by activating autophagy and increasing the probability of random flaps’ survival.

As a better blood-activating agent, NT has been widely used in cerebral infarction, central retinal vein occlusion, and other diseases (Xie et al., 2019; Li H.-L. et al., 2021). Previous studies have shown that NT played a major role in promoting angiogenesis and dilating blood vessels to increase blood flow, but no studies have reported the role of NT in random flaps (Hong et al., 2009; Zheng et al., 2013; Yang et al., 2016; Zhong et al., 2020; Zhu P. et al., 2021). Our results showed that NT could effectively promote the regeneration of skin flap vessels and increased the generation of neovascularization by upregulating the secretion of VEGF and the expression of VE-cadherin to promote the maturation of neovascularization, making it a blood vessel connecting arteries and veins and capable of supplying oxygen.

However, I/R injury is a key problem that cannot be ignored to promote angiogenesis. Reactive oxygen species (ROS) mainly cause the damage of microvessels and parenchymal organs during ischemic tissue reperfusion. The synthesis ability of antioxidant enzymes, which can scavenge free radicals, is impaired in the ischemic tissue, thus aggravating the damage of free radicals to ischemic reperfusion tissue (Neeff et al., 2012; Sun et al., 2014; Szabó et al., 2020). SOD can protect the tissue from ischemia and reperfusion by scavenging free radicals (Ambrosio et al., 1987; Galiñanes et al., 1992; Chen et al., 1996; Zhao et al., 2018). Our experimental results showed that NT could upregulate antioxidant indexes, such as SOD1, eNOS, and HO-1, in tissues and effectively resist I/R injury. These results suggest that NT promotes angiogenesis and regulates the tolerance of random skin flap cells to a harsh environment.

Autophagy is referred to as the rough-surfaced endoplasmic reticulum of the ribosome area of double-membrane package which is part of the cytoplasm and cell organelles. Protein compositions, such as the degradation of domestic demand autophagy, will form, and fusion with lysosomes forming autophagy-lysosome and degradation of the contents of the package to realize the metabolism of the cell itself and some cell organelles needs to be updated (Ariosa et al., 2021; Ganley, 2021; Zhao et al., 2021). In normal cells, small amounts of autophagy are maintained to retain cell homeostasis (Talukdar et al., 2021). Due to the lack of nutrients in random flaps, autophagy is required to decompose damaged organelles to provide energy (Sciarretta et al., 2014; Wu et al., 2020). Therefore, the activation of autophagy in random flaps may improve the survival of random flaps. Our study showed that Beclin-1 protein expression increased in NT-treated flaps, an autophagy agonist, and can activate VPS34 and bind to form the VPS34 complex, promoting the formation of autophagosomes. Our subsequent detection of VPS34 protein expression showed a significant increase in its expression level. LC3 is the gold standard for autophagy expression, and our results showed that the expression of LC3 II/LC3 I in the NT group was significantly increased compared with that in the control group. P62 as a substrate for autophagy was significantly reduced in the NT group.

To further verify that NT promotes the survival of random flaps by activating autophagy, we applied the autophagy inhibitor 3MA to the flaps treated by NT. Experimental results showed that the therapeutic effect of NT was significantly inhibited, and the number of new vessels, blood flow signal intensity, and the survival area of flaps were significantly downregulated. The organizational structure becomes disorganized. Finally, to clarify the pathway of NT’s role in autophagy, the VPS34 inhibitor: VPS34 IN was used to inhibit NT-upregulated VPS34 expression to verify whether NT regulates autophagy through the Beclin-1/VPS34/LC3 pathway. Our results showed that the therapeutic effect of NT decreased significantly after VPS34 IN.

In conclusion, we demonstrate for the first time that NT promotes random flap angiogenesis and improves tissue survival. In addition, NT has been widely used in clinical practice and has high translational significance. Our study confirmed that NT plays a crucial role in promoting angiogenesis and defending against oxidative stress, and this protective effect of NT may be closely related to Beclin-1/VPS34/LC3-mediated autophagy activation.

## Data Availability

The original contributions presented in the study are included in the article/Supplementary Material; further inquiries can be directed to the corresponding authors.
